# Revisiting the Effects of Organized Mammography Programs on Inequalities in Breast Screening Uptake: A Multilevel Analysis of Nationwide Data From 1997 to 2017

**DOI:** 10.3389/fpubh.2022.812776

**Published:** 2022-02-07

**Authors:** Vladimir Jolidon, Vincent De Prez, Piet Bracke, Andrew Bell, Claudine Burton-Jeangros, Stéphane Cullati

**Affiliations:** ^1^Institute of Sociological Research, University of Geneva, Geneva, Switzerland; ^2^Department of Sociology, Ghent University, Ghent, Belgium; ^3^Sheffield Methods Institute, University of Sheffield, Sheffield, United Kingdom; ^4^Population Health Laboratory, University of Fribourg, Fribourg, Switzerland; ^5^Department of Readaptation and Geriatrics, University of Geneva, Geneva, Switzerland

**Keywords:** socioeconomic inequalities, Switzerland, breast cancer screening, marital status, multilevel analysis, organized population-based screening programs

## Abstract

This study revisits the effects of mammography screening programs on inequalities in breast screening uptake in Switzerland. The progressive introduction of regional mammography programs by 12 out of the 26 Swiss cantons (regions) since 1999 offers an opportunity to perform an ecological quasi-experimental study. We examine absolute income and marital status inequalities in mammography uptake, and whether the cantons' implementation of mammography programs moderate these inequalities, as previous research has devoted little attention to this. We use five waves of the Swiss Health Interview Survey covering the 1997–2017 period and comprising data on 14,267 women aged 50–70. Both up-to-date and ever-screening outcomes are analyzed with multilevel models which assess the mammography programs' within-canton effect. Findings show that higher income women and married women (compared to unmarried women) had significantly higher mammography uptake probabilities. Mammography programs did not moderate absolute income differences in up-to-date screening; however, they were associated with smaller absolute income differences in ever-screening uptake. Mammography programs related to higher screening uptake for married women, more than for unmarried women. In conclusion, we showed absolute income inequalities in mammography uptake which were not revealed by previous studies using relative inequality measures. Mammography programs may have contributed to reducing income inequalities in ever-screening, yet this was not observed for up-to-date screening. This study has implication for preventive health interventions—e.g., cancer screening promotion should pay attention to women's marital status since screening programs may widen the screening gap between married and unmarried women.

## Introduction

Socioeconomic inequalities in breast cancer screening uptake have been shown to be higher in countries which do not have a nationwide population-based screening program (1, 2). However, country-specific studies have brought mixed evidence on screening programs' potential to reduce inequalities in mammography uptake (3–8). This is the case of Switzerland where regional mammography programs were not found to importantly moderate socioeconomic inequalities in screening uptake (9).

In Switzerland, 12 out of the 26 cantons (regions) have implemented organized mammography programs at different timepoints from 1999 to present. There is no nationwide mammography program since the cantons autonomously manage their own healthcare system and prevention. In cantons where a program is implemented, eligible women are systematically invited for a mammography every 2 years; while in cantons without a program, screening uptake is “opportunistic”, i.e., it depends on women's individual initiative to undergo mammography and on doctors' recommendation to patients. The Swiss context provides an opportunity to study screening uptake inequalities across organized and opportunistic screening contexts (cantons), in an “ecological quasi-experimental” setting (9). That is, in opportunistic screening contexts, individual factors carry more weight and differences in socioeconomic status may lead to larger screening inequalities, while screening programs may reduce the role of individual economic or social support resources in screening uptake (1).

Inequalities in cancer screening uptake persist since people who possess more resources, such as knowledge, money and social networks, are able to deploy these to adopt available protective strategies and enhance their access to healthcare, while more disadvantaged individuals with less resources face more barriers in accessing healthcare (10). Income level was shown to be an essential determinant of (preventive) health services use across European countries (11, 12). Lower income households are deterred from healthcare uptake by healthcare direct costs, by indirect costs, such as transportation and medication co-payment, as well as opportunity costs from time off work. As a socioeconomic status indicator, income level not only captures individuals' ability to access healthcare, it also accounts for individuals' broader material circumstances which, in turn, impact their psychosocial resources and ability to adopt health-enhancing lifestyles and choices.

It is particularly relevant to assess income-based inequalities in mammography screening uptake in Switzerland, and whether mammography programs moderate these inequalities, since the Swiss health insurance system involves considerable patient out-of-pocket payments which were shown to cause inequalities in healthcare access, as well as healthcare forgoing among lower income populations (13). This context may have contributed to shaping income-inequalities which were documented in cervical and colorectal cancer screening uptake (14–16). Concerning mammography, it is important to note that mammograms conducted within a screening program framework are reimbursed by the national health insurance system, while in cantons without a mammography program reimbursement is subject to doctor's prescription and insurance deductibles involving out-of-pocket expenses. Previous studies focused on relative measurements of screening inequalities in Switzerland, e.g., by comparing income quintiles (9), similarly to the health inequalities literature which has predominantly focused on relative health inequalities (17). However, research suggests that socioeconomic inequalities should also be assessed with absolute measurements, particularly since relative measures do not capture information on absolute variation of prevalence within groups, and can be sensitive to an outcome's overall prevalence (3, 4, 18).

Additionally, we examine how mammography programs moderate the association between marital status and screening uptake. Supportive ties and close relationships are essential determinants of health behaviors and preventive health services uptake (19). Social relations can provide tangible support to access healthcare, as well as encouragement to undergo screening. In particular, a (marital) relationship can have direct positive effects on health status and healthcare uptake. Such health benefit was widely documented and related, in part, to the health-related social control provided by a (marital) partner (20). Thus, the presence of a spouse or a partner is a key social support resource for preventive health behaviors. Marital status was commonly used as a proxy for the support provided by an intimate relationship and being married was found to be associated with higher mammography uptake (21, 22). Nevertheless, little attention was devoted to the effect of marital status on screening uptake in Switzerland, and to how mammography programs may modify this effect.

This study covers a 20-year period from 1997 to 2017. It is the first to analyze the most recent 2017 wave of the Swiss Health Interview Survey in relation to mammography screening, and the most recent mammography programs implemented by three cantons in the 2012–2017 period. We use multilevel modeling to account for the mammography programs' progressive implementation by the Swiss cantons over time. These models allow to assess a context-level variable, such as cantons' mammography program, and how it moderates individual-level variables with cross-level interactions. However, previous studies did not use multilevel models to analyze the mammography programs' moderation effect on screening inequalities across the Swiss cantons.

## Data and Methods

We used data from the Swiss Health Interview Survey (SHIS), a cross-sectional nationally representative survey implemented every 5 years and based on a stratified random selection of residents older than 14 years of age. We pooled the 5 most recent survey waves (from 1997 to 2017) and restricted the sample to 50–70 year-old women (*N* = 17,038). The final sample contained 14,267 women after excluding missing data.

We computed two binary outcomes: “ever-screening” (1 = ever did a mammography, 0 = never did a mammography) and “up-to-date screening” (1 = did a mammography in the past 2 years, 0 = more than 2 years ago). We measured the implementation of mammography screening programs across cantons and time with a variable coded as: (1) “program,” if a canton had implemented a mammography program before a SHIS survey year, and (0) “no program,” if no program was implemented (Supplementary Table S1). Individual-level predictors are monthly household income and marital status,^1^ and control variables are education level, employment status, urban/rural area of residence, linguistic region of residence, age, self-rated health, and general practitioner or gynecologist visits in the last 12 months. We also included dummy variables for survey years and cantons to control for country-wide temporal trends and between-canton unobserved heterogeneity (23). In order to assess absolute income inequalities in screening uptake, we used a continuous measure of monthly household income, as provided by the SHIS. The household income variable is weighted according to the OECD-modified scale and logged. The OECD-modified scale assigns a weight to each household member in order to take into account differences in household size and composition (24): 1.0 to the first adult of the household, 0.5 to each additional household member aged 14 and over, and 0.3 to children under 14 years old. Total household income is divide by the sum of the weights. Descriptive statistics are reported in Supplementary Table S2.

We performed multilevel logistic models with individuals (level-1) nested in canton-year clusters (level-2), i.e., each level-2 cluster combines a specific canton and survey year. The 5 pooled survey waves provided 130 canton-year clusters (26 cantons × 5 survey waves) (Supplementary Table S3). By including a random intercept for canton-year, the multilevel design accounts for similarities between women who belong to the same canton-year cluster and the fact that cantons implemented mammography programs at different timepoints between 1997 and 2017. It also takes into account cluster-level heteroscedasticity and error correlation in data affected by hierarchies, which is not accounted for in standard regression models (25).

First, we used a likelihood ratio test to assess whether models with a random intercept for canton-year performed better than models without random intercepts. Second, we analyzed the effect of household income, marital status and mammography programs on screening uptake (model 1), controlling for all individual-level covariates, and for time and canton fixed effects (dummy variables) to account for country-wide temporal trends and within-canton clustering. Third, we performed cross-level interactions between mammography program and individual-level variables in separate models (models 2a and 2b) to examine whether these programs moderated individual-level differences in screening uptake. The data is analyzed with Stata 16.

## Results

The models with a random intercept for canton-year performed better than equivalent single-level models [up-to-date screening: $\chi_{\left(1\right)}^{2}$ = 1,447.52, *p* < 0.001; ever-screening: $\chi_{\left(1\right)}^{2}$ = 1931.53, *p* < 0.001], revealing significant differences in up-to-date and ever-screening uptake between canton-year clusters. As expected, women with higher household income and married women (compared to unmarried women) had significantly higher up-to-date screening and ever-screening probabilities (Table 1; Supplementary Table S4). Cantons with a mammography program had higher up-to-date screening uptake than cantons without programs, but did not have higher ever-screening uptake.

**Table 1:** Association of up-to-date and ever-screening mammography uptake with individual-level and mammography program variables, results of logistic multilevel analysis, odds ratios and confidence intervals (*n*_individual_ = 14,267; *n*_canton−year_ = 130).

	**Up-to-date screening**	**Ever-screening**
	**OR (95% CI)**	**OR (95% CI)**
**Model 1** ^a^		
**Individual level**		
Household income (logged)	1.222^***^ (1.130–1.321)	1.225^***^ (1.128–1.331)
Marital status (ref: single, divorced, widow) married	1.364^***^ (1.261–1.475)	1.428^***^ (1.305–1.562)
**Canton-year level**		
Mammography program (ref: no program) program	1.737^***^ (1.463–2.062)	1.170 (0.961–1.424)
**Level-2 variance**		
Canton-year^b^	0.015	0.015

Significance levels: *p ≤ 0.05, **p ≤ 0.01,

*** *p ≤ 0.001*.

a *Model adjusted for level-1 (individual) covariates (including education level, employment status, age, linguistic region, area of residence, self-rated health, GP or gynecologist visits in the past 12 months), and time (survey year dummies) and canton-level heterogeneity (canton dummies) at the model's level-2*.

b *The inclusion of a level-2 (canton-year) variance in the models was assessed with likelihood ratio tests. The tests showed significant differences between canton-year clusters in up-to-date [$\chi_{\left(1\right)}^{2}$ = 7.44, p = 0.006] and ever-screening [$\chi_{\left(1\right)}^{2}$ = 3.92, p = 0.05] uptake*. *Between 1997 and 2017, 12 out of 26 cantons implemented mammography programs*. *Source: SHIS 1997–2017*.

**Figure 1:**
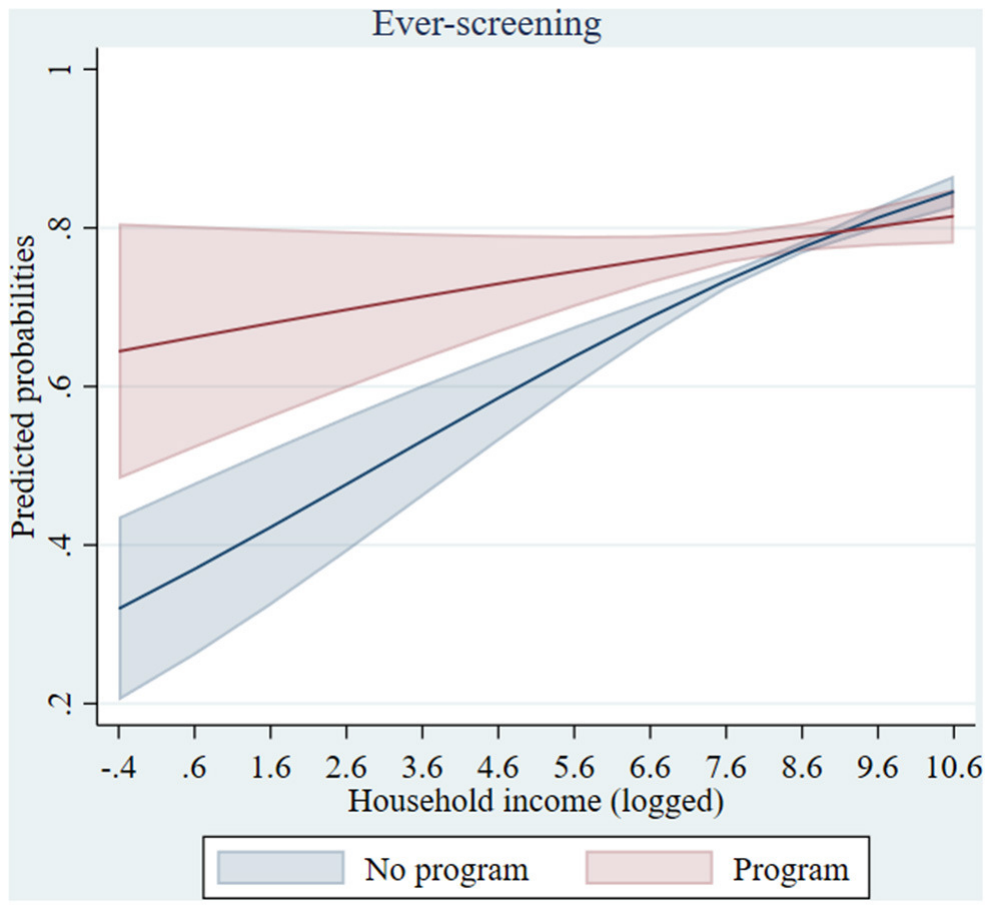
Predicted probabilities of mammography ever-screening by household income in cantons with/without a mammography program (based on model 2a). Confidence intervals were calculated using a multiplier of 1.39 standard errors since two parameters are compared rather than a parameter and a single (fixed) point (Goldstein H, Healy MJR. The graphical presentation of a collection of means. *J R Statist Soc*. (1995) 158:175–7).

**Figure 2:**
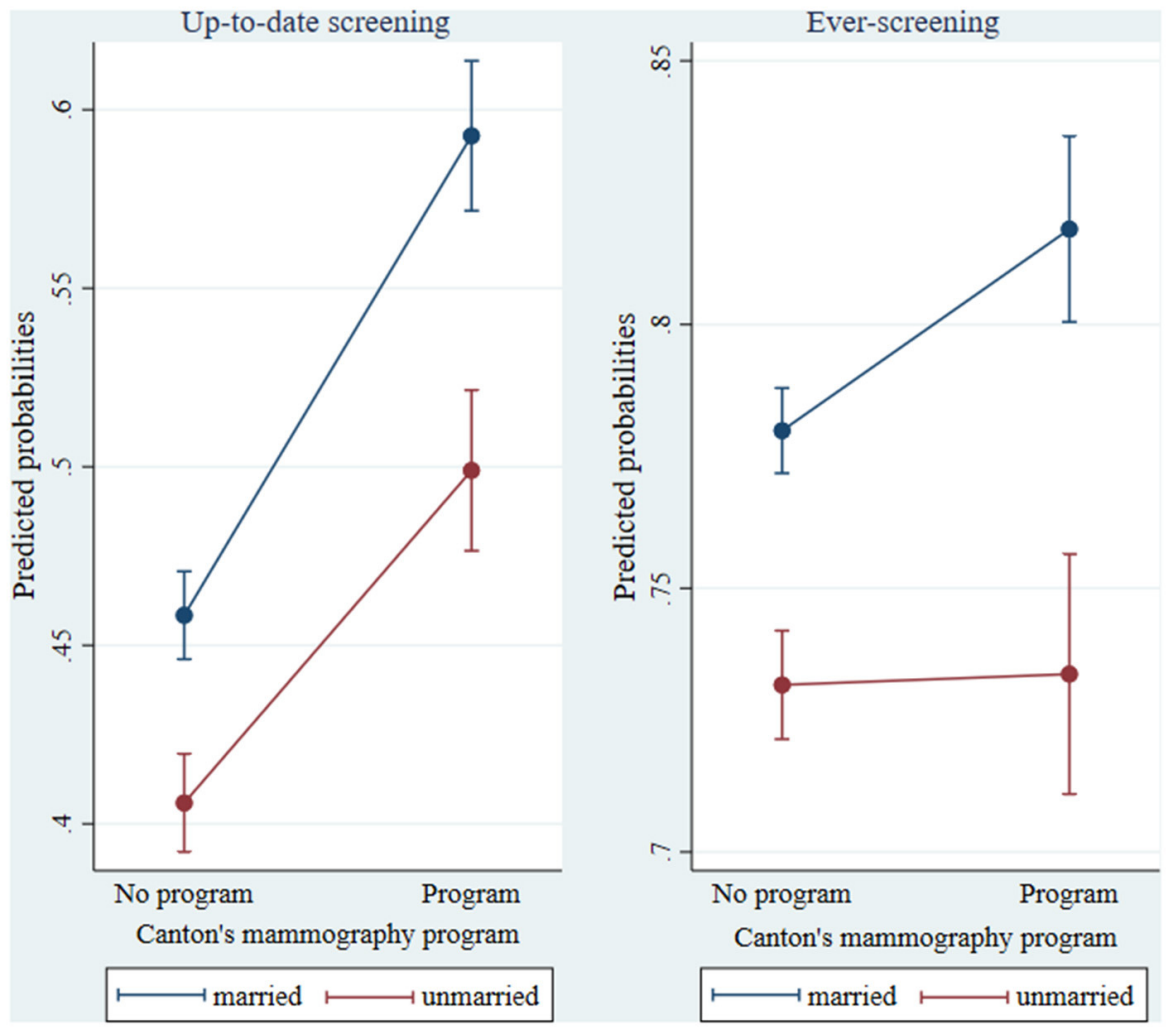
Predicted probabilities of mammography up-to-date and ever-screening by marital status in cantons with/without a mammography program (based on model 2b). Confidence intervals were calculated using a multiplier of 1.39 standard errors since two parameters are compared rather than a parameter and a single (fixed) point (Goldstein H, Healy MJR. The graphical presentation of a collection of means. *J R Statist Soc*. (1995) 158:175–7).

As shown in Table 2, mammography programs were associated with smaller income differences in ever-screening, as pointed out by the cross-level interaction term significant at a 95% confidence level (Supplementary Table S5). This effect is graphed in Figure 1 which shows that women with lower household income had higher ever-screening probabilities in cantons with a mammography program, than in cantons without a program. It was significant for women with monthly household income from the first to the fifth decile, i.e., for women with monthly household income lower than 4,000 CHF,^2^ and the lower the income the stronger the effect was, as analyses presented in Supplementary Material revealed (Supplementary Table S6). For up-to-date screening, no interaction effect was observed. Finally, a cross-level interaction also revealed that, in cantons with a mammography program, married women had higher screening uptake than their unmarried counterparts, compared to cantons without a program (Table 2). Figure 2 depicts this effect for both up-to-date and ever-screening uptake.

**Table 2:** Cross-level interactions between mammography program and individual-level variables in their effect on mammography uptake, results of logistic multilevel analysis, odds ratios and confidence intervals (*n*_individual_ = 14,267; *n*_canton−year_ = 130).

	**Model 2a**		**Model 2b**	
	**Up-to-date screening**	**Ever-screening**	**Up-to-date screening**	**Ever-screening**
	**OR (95% CI)**	**OR (95% CI)**	**OR (95% CI)**	**OR (95% CI)**
**Individual level**				
Household income	1.233^***^ (1.116–1.363)	1.302^***^ (1.175–1.443)	1.223^***^ (1.131–1.322)	1.228^***^ (1.130–1.334)
Marital status (ref: single, divorced, widow)	1.363^***^ (1.261–1.475)	1.426^***^ (1.303–1.560)	1.283^***^ (1.169–1.409)	1.357^***^ (1.229–1.498)
**Canton-year level**				
Mammography program (ref: no program) program	2.092 (0.626–6.994)	4.716^*^ (1.189–18.702)	1.551^***^ (1.275–1.887)	1.012 (0.806–1.272)
**Cross-level interaction**				
Household income (logged) × program	0.978 (0.846–1.130)	0.844^*^ (0.715–0.996)		
Married × program			1.214^*^ (1.033–1.426)	1.299^*^ (1.047–1.612)
**Level-2 variance**				
Canton-year^a^	0.015	0.015	0.015	0.015

*Significance levels:*

* *p ≤ 0.05,*

**p ≤ 0.01,

*** *p ≤ 0.001*.

a *The inclusion of a level-2 (canton-year) variance in the models was assessed with likelihood ratio tests. The tests showed significant differences between canton-year clusters in Model 2a for up-to-date [$\chi_{\left(1\right)}^{2}$ = 7.37, p = 0.007] and ever-screening [$\chi_{\left(1\right)}^{2}$ = 3.86, p = 0.05] uptake, and in Model 2b for up-to-date [$\chi_{\left(1\right)}^{2}$ = 7.09, p = 0.008] and ever-screening [$\chi_{\left(1\right)}^{2}$ = 3.92, p = 0.05] uptake*. *Models are adjusted for all level-1 (individual) covariates, and time (survey year dummies) and canton-level heterogeneity (canton dummies) at the model's level-2*. *Source: SHIS 1997–2017*.

## Discussion

We found evidence of absolute income inequalities in mammography uptake in Switzerland over the 1997–2017 period. The fact that mammograms are reimbursed by the health insurance system in cantons with screening programs, while more constraints (e.g., doctor's prescription) and out-of-pocket expenses apply in cantons without programs, might be driving these income-based inequalities. These absolute inequalities were not evidenced by Cullati et al. (9) who examined relative income inequalities. While relative measures focus on the magnitude of inequalities between groups, our results showed that absolute screening inequalities persisted across the entire range of income levels. Thus, it is recommended to consider both absolute and relative measures to accurately monitor screening inequalities and inform policymakers (3, 4, 17).

The cantons' implementation of mammography programs was associated with higher up-to-date screening uptake; however, we found no evidence that these programs moderated income inequalities in up-to-date screening. This confirms the findings of Cullati et al. (9) who did not observe a reduction of up-to-date income-based screening inequalities associated with mammography programs over the 1992–2012 period. In contrast, European cross-national research evidenced that screening programs contributed to reducing inequalities in screening uptake (2). This was explained by the screening programs' systematic invitation of all eligible women which offers equal access to screening and reduces the information gap between individuals with different socioeconomic status and health literacy levels.

Persistence of the observed screening inequalities in Switzerland might be explained, partly, by the fact that not all women undergo mammography through the program in cantons with organized screening, and opportunistic screening uptake may still persist. Furthermore, inequalities in screening knowledge and negative attitudes toward mammography may persist, and particularly affects lower socioeconomic groups' screening uptake (26, 27). While research showed that screening programs successfully increase overall screening participation, and that invitation letters importantly contribute to this increase, they may not necessarily reduce inequalities in screening uptake (28, 29). Program invitation mechanisms and invitation letter features may affect participation. For example, long and detailed letters can discourage lower socioeconomic status individuals to participate, and whether a scheduled appointment is included in the letter was also shown to have an impact on screening participation (30). Information on the features and specific content of invitation letters sent by the cantons' programs should be collected (for example, by Swiss Cancer Screening in the monitoring report of the mammography programs) so future research may assess their effects in order to inform interventions. Finally, we should note that the majority of the mammography programs were implemented recently, in the last 10 years of the 20-year period considered in this study. More time might be necessary to be able to observe an inequality-reducing effect, particularly since higher socioeconomic status individuals are usually quicker than more disadvantaged individuals in adopting the offer of new preventive health services (31).

Mammography program implementation was not associated with significantly higher ever-screening uptake. However, it was associated with smaller income inequalities in ever-screening. As depicted in Figure 1, ever-screening was higher among lower income women in cantons with organized screening. Previous studies suggested that the reduction of financial barriers and awareness-raising brought by the Swiss mammography programs may have played a role in promoting uptake among lower income women who had never screened (without necessarily increasing overall ever-screening uptake) (5, 32). Our results highlighted that not only “up-to-date screeners” but also “ever- and never-screeners” should be taken into account, as different determinants may shape their screening uptake. Notably, never-screeners were shown to face greater socioeconomic barriers to cervical cancer screening uptake (16) and to be more strongly affected by a lack of screening knowledge in their mammography uptake (33, 34). Cognitive, emotional, structural and communication barriers might differ between never-screeners and those who have already screened but are off-schedule (33). Thus, further studies are needed to investigate the specific reasons for non-attendance in different groups and tailor interventions according to screening status (35). Otherwise, standard invitations and reminder letters may fail to trigger participation among participants with specific profiles.

Married women had a higher mammography uptake than unmarried women and some evidence indicated that this gap increased in cantons with organized programs, compared to cantons without programs. Spouses can encourage each other's health-enhancing behaviors, and a couple's shared psychosocial and economic resources may also facilitate healthcare use (22, 36). Oppositely, those who do not have a partner are more at risk of delaying contacts with healthcare services. Having a marital partner may help mitigate well-known barriers to cancer screening, such as fear or embarrassment, and may provide the practical support facilitating healthcare access and screening program utilization. This finding is important to physicians who should pay more attention to the screening uptake of women living alone or without a (marital) partner.

This study has strengths and limitations. Our statistical models preclude causal inference. However, controlling for relevant confounders of mammography uptake provides support to a causal interpretation of the mammography programs' effect. Moreover, by using a multilevel model which controlled for country-wide temporal trends and canton-level unobserved heterogeneity, we extend previous studies and assessed the mammography programs' within-canton, between-canton-year effect. We used marital status as a proxy for the social support provided by an intimate relationship since information on respondents' partnership and cohabitation status was not available across the 5 waves of the SHIS. The marital status variable does not capture the social support provided by non-married and homosexual relationships and may involve some misclassification bias. Finally, survey data are susceptible to errors in self-reporting, and confounding from unmeasured variables cannot be excluded.

To conclude, this study reported evidence of absolute income inequalities in mammography uptake in Switzerland. Mammography programs may have contributed to reducing these income inequalities in ever-screening uptake; however, programs have not modified inequalities in up-to-date screening, and may have potentially increased inequalities in screening uptake between married and unmarried women. Hence, more specific and targeted public health interventions might be required to complement mammography programs and better reach women with lower income and those who do not live with a (marital) partner, and support their screening uptake. That is, the cost-reducing intervention of screening programs may not suffice to engage specific groups of non-participants in mammography screening and further strategies should be considered (28).

Interventions focusing on behavioral change can be successful. For example, text message reminders can be a cost-effective solution which was shown to increase mammography uptake among women who never had a mammography and hard-to-reach populations (37). Providing a phone number in the invitation letter to a call center with patient navigators who schedule appointments and are able to give information and tackle (structural and psychological) barriers to screening was also shown to improve mammography uptake among socioeconomically disadvantaged women (38). Provision of information through invitation letters and their effect on screening uptake may reach a limit when program coverage is high (39). However, combining postal letters with other invitation strategies, such as phone calls or text messages, in a “multiple-component intervention”, was shown to be effective to increase uptake among low income women (40). Finally, there is overwhelming evidence that recommendation from primary care physician to patient improves screening uptake (41). Their involvement in screening programs should thus be promoted. Beyond the mere recommendation to undergo screening, patient-provider communication is fundamental. As studies showed, physician enthusiasm and encouragement perceived by patients is a key determinant of screening adherence (41), and a more comprehensive patient-provider communication on broader topics including sexual health can improve breast and cervical screening uptake among unmarried women (42).

Breast cancer is a leading cause of women's amenable mortality in Switzerland, where both geographic and socioeconomic inequalities in breast cancer care and stage at diagnosis were documented (43). Switzerland's healthcare system weakness in tackling health inequalities, implementing health prevention and producing nationwide health data and quality of care indicators was linked to its high decentralization (due to the cantons' autonomy to manage healthcare) (44, 45). Regional mammography programs may thus risk reproducing socioeconomic and geographic inequalities in breast cancer outcomes. For these reasons, we join previous research in stressing the need for more and better nationwide coordination of quality-controlled prevention and cancer screening programs in Switzerland to reduce disparities in early detection (46).

## Data Availability Statement

This study used data from the Swiss Health Interview Survey. The data is available for a fee (1600 Swiss Francs, plus 7.7% tax) and users must request permission from the Swiss Federal Statistical Office (sgb@bfs.admin.ch). Data must be destroyed after five years. Requests to access these datasets should be directed to Swiss Federal Statistical Office (sgb@bfs.admin.ch).

## Ethics Statement

The research uses anonymized survey data from the Swiss Health Interview Survey (SHIS). Access to the data was granted by the Swiss Federal Statistical Office upon application and contract signing. Compliance with Swiss National Guidelines and Legal Basis (Swiss statistical law) were stated in the data delivery contract signed with the Swiss Federal Statistical Office.

## Author Contributions

VJ: conceptualization, methodology, formal analysis, writing—original draft, and writing—review and editing. VD and AB: conceptualization and writing—review and editing. PB, CB-J, and SC: conceptualization, writing—review and editing, supervision, funding acquisition, and project administration. All authors approved the final version of the manuscript.

## Funding

This research was conducted within the project PREVENT—Social inequalities in cancer prevention care and fundamental social causes: a comparative study of innovative technologies and (in)effective policies which was funded by the Research Foundation—Flanders (Grant Number FWOOPR2018005701) and the Swiss National Science Foundation (Grant Number 176115). Open access publication fees were covered by the Swiss National Science Foundation. The funding bodies had no involvement in the study design, the collection, analysis, and interpretation of data, the writing of the manuscript, nor in the decision to submit the manuscript for publication.

## Conflict of Interest

The authors declare that the research was conducted in the absence of any commercial or financial relationships that could be construed as a potential conflict of interest.

## Publisher's Note

All claims expressed in this article are solely those of the authors and do not necessarily represent those of their affiliated organizations, or those of the publisher, the editors and the reviewers. Any product that may be evaluated in this article, or claim that may be made by its manufacturer, is not guaranteed or endorsed by the publisher.

## Footnotes

^1^We used the marital status since information on respondents' partnership and co-habitation status was not available across the 5 survey waves of the SHIS, but only in the 3 most recent waves (2007–2017 period). Analysis of those 3 waves using the partnership status variable (whether or not a respondent is “living with a partner”) instead of marital status produced substantively similar results.

^2^Monthly household income was weighted using the OECD-modified scale, as detailed in the methods section. 1 CHF was approximately equivalent to 0.79 USD in average over the period considered by this study (1997–2017).
