# Supplementary material for: Revisiting the Effects of Organized Mammography Programs on Inequalities in Breast Screening Uptake: A Multilevel Analysis of Nationwide Data From 1997 to 2017
Source: Front Public Health. 2022 Feb 7;10:812776. doi: 10.3389/fpubh.2022.812776 (PMC8858931; doi:10.3389/fpubh.2022.812776)
Supplement: Supplementary file 1 [file Data_Sheet_1.pdf]

Table S.1 Mammography screening programmes implemented in Swiss cantons at the time of a Swiss Health Interview Survey (SHIS) survey wave

Canton / SHIS survey year	1997	2002	2007	2012	2017
ZH	0	0	0	0	0
BE	0	0	0	0	1
LU	0	0	0	0	0
UR	0	0	0	0	0
SZ	0	0	0	0	0
OW	0	0	0	0	0
NW	0	0	0	0	0
GL	0	0	0	0	0
ZG	0	0	0	0	0
FR	0	0	1	1	1
SO	0	0	0	0	0
BS	0	0	0	0	1
BL	0	0	0	0	0
SH	0	0	0	0	0
AR	0	0	0	0	0
AI	0	0	0	0	0
SG	0	0	0	1	1
GR	0	0	0	1	1
AG	0	0	0	0	0
TG	0	0	0	1	1
TI	0	0	0	0	1
VD	0	1	1	1	1
VS	0	1	1	1	1
NE	0	0	0	1	1
GE	0	1	1	1	1
JU	0	0	1	1	1

Note: (0) = no mammography screening programme; (1) = mammography screening programme implemented before the SHIS survey wave

Table S.2 Descriptive statistics for 50 to 70 year-old women in the Swiss Health Interview Survey (SHIS) waves, N = 14,267

	1997		2002		2007		2012		2017	
	N	*%*	*N*	*%*	*N*	*%*	*N*	*%*	*N*	*%*
Dependent variables										
Up-to-date screening										
did a mammography more than 2 years ago	1207	69.93	1313	47.75	1512	52.63	1707	50.77	1759	49.47
did a mammography in the past 2 years	519	30.07	1437	52.25	1361	47.37	1655	49.23	1797	50.53
Ever-screening										
never did a mammography	717	41.54	739	26.87	611	21.27	655	19.48	617	17.35
ever did a mammography	1009	58.46	2011	73.13	2262	78.73	2707	80.52	2939	82.65
Independent variables (categorical)										
Education										
primary	550	31.87	629	22.87	673	23.42	682	20.29	640	18.00
upper-secondary	1067	61.82	1874	68.15	1757	61.16	1994	59.31	2068	58.16
tertiary	109	6.32	247	8.98	443	15.42	686	20.40	848	23.85
Employment status										
non employed	1026	59.44	1397	50.80	1381	48.07	1316	39.14	1371	38.55
full-time	263	15.24	489	17.78	495	17.23	654	19.45	698	19.63
part-time	437	25.32	864	31.42	997	34.70	1392	41.40	1487	41.82
Marital status										
unmarried (single, divorced, widow)	818	47.39	1215	44.18	1348	46.92	1231	36.62	1224	34.42
married	908	52.61	1535	55.82	1525	53.08	2131	63.38	2332	65.58
Linguistic region										
German	1040	60.25	1795	65.27	1717	59.76	2192	65.20	2337	65.72
French	513	29.72	758	27.56	890	30.98	921	27.39	942	26.49
Italian	173	10.02	197	7.16	266	9.26	249	7.41	277	7.79
Area of residence										
urban	1274	73.81	1962	71.35	2047	71.25	2420	71.98	2347	66.00

rural	452	26.19	788	28.65	826	28.75	942	28.02	1209	34.00
Number of GP and gynaecologist visits in the past 12 months										
2+	1251	72.48	1677	60.98	1777	61.85	2157	64.16	2299	64.65
1	226	13.09	527	19.16	576	20.05	627	18.65	703	19.77
0	249	14.43	546	19.85	520	18.10	578	17.19	554	15.58
	Mean	*SD*	*Mean*	*SD*	*Mean*	*SD*	*Mean*	*SD*	*Mean*	*SD*
Independent variables (continuous)										
Income (logged)	8.08	0.47	8.23	0.49	8.27	0.46	8.30	0.49	8.27	0.68
Age	59.64	6.15	59.36	5.86	59.98	5.94	59.04	6.15	59.29	6.01
Self-rated health	2.90	0.81	2.96	0.74	2.97	0.72	3.05	0.85	3.11	0.83

Table S.3 Number of 50 to 70 year-old women per canton-year combinations in the Swiss Health Interview Survey (SHIS) waves 1997, 2002, 2007, 2012 and 2017

Canton / SHIS survey year	1997	2002	2007	2012	2017	Total
ZH	204	255	380	335	352	1,526
BE	233	242	353	284	302	1,414
LU	107	132	156	187	158	740
UR	6	25	59	77	103	270
SZ	25	18	64	72	153	332
OW	3	20	8	6	92	129
NW	7	22	9	8	12	58
GL	12	34	7	8	11	72
ZG	22	118	23	165	23	351
FR	39	122	137	164	175	637
SO	37	138	62	39	62	338
BS	30	156	50	162	34	432
BL	49	152	85	140	151	577
SH	14	42	29	15	12	112
AR	8	28	78	72	120	306
AI	1	6	4	2	5	18
SG	63	131	98	86	165	543
GR	58	50	31	106	172	417
AG	117	142	207	198	196	860
TG	28	46	45	180	168	467
TI	168	192	262	242	263	1,127
VD	138	163	222	283	281	1,087
VS	137	125	159	172	176	769
NE	49	119	109	99	113	489
GE	153	181	169	184	169	856
JU	18	91	67	76	88	340
Total	1,726	2,750	2,873	3,362	3,556	14,267

Table S.4 Association of up-to-date and ever-screening mammography uptake with individual-level and mammography programme variables, results of logistic multilevel analysis, odds ratios and confidence intervals ($n_{individual}$ = 14,267; $n_{canton\text{-}year}$ = 130)

	Up-to-date screening	Ever-screening
	OR (95% CI)	OR (95% CI)
**Model 1**[a]		
Individual level		
Household income (logged)	1.222***(1.130-1.321)	1.225***(1.128-1.331)
Marital status (ref: single, divorced, widow)		
married	1.364***(1.261-1.475)	1.428***(1.305-1.562)
Education (ref: primary)		
upper-secondary	1.067(0.970-1.173)	1.150*(1.033-1.280)
tertiary	0.992(0.870-1.131)	1.091(0.936-1.271)
Employment status (ref: non employed)		
full-time	1.083(0.959-1.223)	1.078(0.937-1.240)
part-time	1.065(0.968-1.172)	1.115(0.998-1.245)
Linguistic region (ref: German)		
French	2.079***(1.674-2.583)	2.323***(1.747-3.088)
Italian	2.513*(1.210-5.221)	2.631*(1.061-6.525)
Area of residence (ref: urban)		
rural	0.877**(0.799-0.962)	0.769***(0.692-0.854)
Number of GP and gynaecologist visits in the past 12 months (ref: 2+)		
1	0.271***(0.243-0.303)	0.375***(0.337-0.418)
0	0.653***(0.593-0.720)	0.665***(0.594-0.744)
Self-rated health (continuous)	0.997(0.949-1.047)	0.956(0.902-1.013)
Age (continuous)	0.998(0.991-1.006)	1.029***(1.020-1.037)
Canton-year level		
Mammography programme (ref: no programme)		
programme	1.737***(1.463-2.062)	1.170(0.961-1.424)
Level-2 variance		
Canton-year[b]	0.015	0.015

Significance levels: *p ≤ 0.05, **p ≤ 0.01, ***p ≤ 0.001

[a]Model adjusted for time (survey year dummies) and canton-level heterogeneity (canton dummies) at the model's level-2.

[b]The inclusion of a level-2 (canton-year) variance in the models was assessed with likelihood ratio tests. The tests showed significant differences between canton-year clusters in up-to-date ($\chi2(1)=7.44$, p=0.006) and ever-screening ($\chi2(1)=3.92$, p=0.05) uptake.

Note: Between 1997 and 2017, 12 out of 26 cantons implemented mammography programmes.

Source: SHIS 1997-2017

Table S.5 Cross-level interactions between mammography programme and individual-level variables in their effect on mammography uptake, results of logistic multilevel analysis, odds ratios and confidence intervals ($n_{individual}$ = 14,267; $n_{canton\text{-}year}$ = 130)

	**Model 2a**		**Model 2b**	
	Up-to-date screening	Ever-screening	Up-to-date screening	Ever-screening
	OR (95% CI)	OR (95% CI)	OR (95% CI)	OR (95% CI)
Individual level				
Household income	1.233***(1.116-1.363)	1.302***(1.175-1.443)	1.223***(1.131-1.322)	1.228***(1.130-1.334)
Marital status (ref: single, divorced, widow)				
married	1.363***(1.261-1.475)	1.426***(1.303-1.560)	1.283***(1.169-1.409)	1.357***(1.229-1.498)
Education (ref: primary)				
upper-secondary	1.066(0.969-1.172)	1.143*(1.027-1.273)	1.067(0.970-1.173)	1.150*(1.033-1.280)
tertiary	0.991(0.870-1.130)	1.084(0.930-1.263)	0.991(0.869-1.130)	1.09(0.935-1.269)
Employment status (ref: non employed)				
full-time	1.083(0.959-1.223)	1.076(0.935-1.237)	1.082(0.958-1.222)	1.077(0.936-1.239)
part-time	1.066(0.968-1.173)	1.116(1.000-1.246)	1.065(0.968-1.172)	1.114(0.998-1.244)
Linguistic region (ref: German)				
French	2.080***(1.674-2.583)	2.326***(1.750-3.092)	2.087***(1.680-2.593)	2.330***(1.752-3.099)
Italian	2.513*(1.210-5.219)	2.633*(1.060-6.538)	2.548*(1.227-5.292)	2.671*(1.079-6.613)
Area of residence (ref: urban)				
rural	0.877**(0.799-0.962)	0.770***(0.693-0.856)	0.877**(0.800-0.962)	0.770***(0.693-0.855)
Number of GP and gynaecologist visits in the past 12 months (ref: 2+)				
1	0.271***(0.243-0.303)	0.375***(0.336-0.418)	0.270***(0.242-0.302)	0.374***(0.336-0.417)
0	0.653***(0.593-0.720)	0.665***(0.594-0.744)	0.654***(0.594-0.720)	0.665***(0.594-0.744)
Self-rated health (continuous)	0.997(0.949-1.047)	0.954(0.900-1.012)	0.996(0.949-1.046)	0.955(0.901-1.013)
Age (continuous)	0.999(0.991-1.006)	1.029***(1.020-1.038)	0.998(0.991-1.006)	1.029***(1.020-1.037)
Canton-year level				
Mammography programme (ref: no programme)				
programme	2.092(0.626-6.994)	4.716*(1.189-18.702)	1.551***(1.275-1.887)	1.012(0.806-1.272)
Cross-level interaction				
Household income (logged) x programme	0.978(0.846-1.130)	0.844*(0.715-0.996)		
Married x programme			1.214*(1.033-1.426)	1.299*(1.047-1.612)
Level-2 variance				
Canton-year[a]	0.015	0.015	0.015	0.015

Significance levels: *p ≤ 0.05, **p ≤ 0.01, ***p ≤ 0.001

[a]The inclusion of a level-2 (canton-year) variance in the models was assessed with likelihood ratio tests. The tests showed significant differences between canton-year clusters in Model 2a for up-to-date ($\chi2(1)=7.37$, $p=0.007$) and ever-screening ($\chi2(1)=3.86$, $p=0.05$) uptake, and in Model 2b for up-to-date ($\chi2(1)=7.09$, $p=0.008$) and ever-screening ($\chi2(1)=3.92$, $p=0.05$) uptake.

Note: Models are adjusted for time (survey year dummies) and canton-level heterogeneity (canton dummies) at the model's level-2.
Source: SHIS 1997-2017

Table S.6 Difference in the predicted probabilities of mammography ever-screening between cantons with and without a mammography programme by household income deciles

Household income decile	Weighted household income[a] (CHF)	Difference in predicted probabilities	Standard error	83.5% confidence interval[b]
1	2,000	0.041	0.018	[0.017, 0.066]
2	2,533	0.034	0.016	[0.012, 0.057]
3	3,000	0.029	0.015	[0.008, 0.051]
4	3,333	0.026	0.015	[0.005, 0.048]
5	4,000	0.021	0.015	[0.000, 0.042]
6	4,300	0.019	0.015	[-0.001, 0.040]
7	5,000	0.015	0.015	[-0.006, 0.037]
8	5,600	0.012	0.016	[-0.009, 0.034]
9	6,667	0.008	0.016	[-0.015, 0.030]

[a]Monthly household income was weighted using the OECD-modified scale, as detailed in the methods section.
[b]Confidence intervals were calculated using a multiplier of 1.39 standard errors since two parameters are compared rather than a parameter and a single (fixed) point (Goldstein H, Healy MJR. The Graphical Presentation of a Collection of Means. 1995;*J R Statist Soc*.(158):175-177).

Note: Predicted probabilities were calculated based on the cross-level interaction estimated in model 2b for ever-screening uptake.
Source: SHIS 1997-2017
